# Patient experiences in multidisciplinary care for persistent somatic symptoms across four European countries: a cross-sectional comparison

**DOI:** 10.1136/bmjopen-2024-097593

**Published:** 2025-03-05

**Authors:** Nick Mamo, Aleksandra Kustra-Mulder, Denise J C Hanssen, Angelika Weigel, Lineke Tak, Tim C Olde Hartman, Bernd Löwe, Judith G M Rosmalen

**Affiliations:** 1 Alkura Specialist Center Persistent Somatic Symptoms, Dimence Groep, Deventer, Netherlands; 2 Department of Psychiatry, University Medical Centre, Groningen, Netherlands; 3 Department of Psychosomatic Medicine and Psychotherapy, University Medical Center Hamburg-Eppendorf, Hamburg, Germany; 4 Department of Primary and Community Care, Radboud Universitair Medisch Centrum, Nijmegen, Netherlands; 5 Department of Internal Medicine, University Medical Centre, Groningen, Netherlands

**Keywords:** PSYCHIATRY, Patients, Organisation of health services, Cross-Sectional Studies, Chronic Disease

## Abstract

**Abstract:**

**Objectives:**

The aim of this study is to explore patients with persistent somatic symptoms and functional disorders’ (PSS/FD) experiences of and preferences for multidisciplinary care across Europe. A further aim is to compare the experiences of and preferences for multidisciplinary care of patients to those of healthcare professionals (HCPs) in the Netherlands.

**Design:**

Cross-sectional online survey.

**Setting:**

Patients with PSS/FD from across Europe (Germany, Italy, the Netherlands and Poland) and HCPs working in the care for PSS/FD across all levels of care in the Netherlands.

**Primary and secondary outcome measures:**

Outcome measures for both patients and HCPs related to experiences of multidisciplinary care, communication between professionals and patients, as well as the main point of contact for patients.

**Results:**

600 patients responded (Germany: n=198; Italy: n=174; Netherlands: n=137; Poland; n=91), and 152 HCPs responded from the Netherlands. Compared with the other countries, patients from the Netherlands generally received less multidisciplinary care, from fewer disciplines. Regarding most variables related to interprofessional communication, patients in Italy rated their experience significantly better than in most other countries. Generally, patients preferred either their general practitioner (GP) or a medical specialist as their main point of contact, and not mental health professionals. In contrast, HCPs preferred mental health professionals as the main point of patient contact, followed by GPs. In all variables, patients in the Netherlands rated interprofessional communication significantly lower than HCPs in the Netherlands did.

**Conclusions:**

Patients have different experiences of interdisciplinary communication, also reporting lower-quality communication than HCPs, though differences are seen between countries. Future studies should look at the reasons for this and how this can lead to improved care for PSS/FD.

**STRENGTHS AND LIMITATIONS OF THIS STUDY**

- This study compares the experiences of patients with persistent somatic symptoms and functional disorders (PSS/FD) across multiple European countries.
- A number of questions are matched allowing for a comparison of patients with PSS/FD and healthcare professionals (HCPs).
- Convenience sampling was used, which limits the generalisability of the overall results.
- Patients were only involved if they had a certain disease severity, so we are only assessing the experiences and preferences of patients with PSS/FD who are more likely to need complex care.
- HCPs and patients with PSS/FD are not matched—we do not assess here the HCPs treating the responding patients, or whether they were specifically treating the patient’s PSS/FD or another condition.

## Introduction

 Persistent somatic symptoms (PSS) are characterised by physical symptoms persisting for months, regardless of their underlying cause.^1^ Functional disorders (FDs) are a group of disorders recognised by patterns of PSS. These include conditions such as Fibromyalgia, a disorder of chronic widespread pain,^2^ functional neurological disorder, a disorder with impairment in voluntary neurological function^3^ and somatic symptom disorder, a disorder characterised by persistent somatic complaints alongside disproportionate health-related thoughts, feelings and behaviours in relation to these symptoms.^4^ FDs affect multiple body systems and are often influenced by multiple biopsychosocial factors.^5^ In a recent review on FD, collaborative care networks (CCNs) often had seven or eight different disciplines involved in providing care,^6^ with wide variation in the setup of the CCNs and therapies provided. Indeed, due to the symptom persistence and possible involvement of multiple body symptoms and biopsychosocial factors, people suffering from PSS/FD may benefit from multidisciplinary care.^7^

Unfortunately, PSS/FD care is often fragmented, with patients facing the possibility of long care trajectories, which at times fail, leaving them untreated or having to start over.^8 9^ Healthcare professionals (HCPs) from different European countries with different healthcare systems report that better interprofessional collaboration is important for symptom improvement.^10^ Further to this, a review found better symptom outcomes in specialised care settings as opposed to current primary and secondary care,^11^ which suggests that better collaboration and referrals between disciplines and between primary, secondary and more specialised care services is important to improve care outcomes.

Previous studies have described multiple barriers to achieving better interdisciplinary collaboration. From the perspective of HCPs working in the field of PSS/FD, these include such factors as not having access to appropriate care at the right time, issues with the diagnostic processes and recognition of FD diagnoses, a focus on the biomedical approach and resource challenges.^10^ As an illustration, one study found that patients with functional neurological disorder had significantly more problems than those with multiple sclerosis in accessing patient-centred care, relationships with HCPs and accessing community care.^12^ However, we do not know much about the multidisciplinary care experience of patients with PSS/FD, or their preferences, especially across countries and healthcare systems.

Therefore, in this study, we aim to explore patients’ experiences of and preferences for multidisciplinary care across Germany, Italy, The Netherlands and Poland in accessing PSS/FD care. The four countries included are selected due to their diverse health systems, in particular, differing in their funding structures and in access to secondary care.^13^,^16^ In Italy and the Netherlands, general practitioners (GPs) act as gatekeepers, whereas in Germany and Poland, they do not, although GPs are usually the first point of contact in Germany. In terms of funding, Italy has a national health service, while Poland, Germany and the Netherlands have a compulsory insurance-based system. However, these differ with Poland providing a social health insurance system for all providing primary and some secondary care services (alongside high private healthcare use), Germany providing a mixed statutory and private health insurance system, and the Netherlands providing regulated private health insurance.

We also aim to compare how the experiences of and preferences for multidisciplinary care of patients with PSS/FD relate to those of HCPs in the Netherlands. Originally, we aimed to compare patients and HCPs in all four countries; however, with relatively low HCP sample sizes in Germany, Italy and Poland, we felt that we would not be able to draw meaningful conclusions in these countries. The results of this study will allow for recommendations to improve collaborative care for PSS/FD generally and with specific recommendations for these four countries.

## Methods

This study uses survey data from the ARISE project (‘Healthcare Online Survey Europe’—see osf.io/3q6hz, osf.io/rx7t9).^17^ The ARISE project is part of the innovative training network ETUDE (Encompassing Training in fUnctional Disorders across Europe).^18^ The ARISE project aims to identify aspects of healthcare that may influence the symptom course of individuals with or at risk for PSS. Also, this project aims to improve our understanding of patients’ and HCPs’ perspectives on healthcare for PSS across Europe. This study was preregistered on Open Science Forum (OSF): osf.io/rybue (see also online supplemental material). In the current study, we aimed to understand multidisciplinary care experiences by looking at communication and language use between patients with PSS/FD and HCPs, as well as satisfaction of patients with healthcare providers. Where this paper refers to multidisciplinary care, this does not imply team-based care, only that multiple disciplines were involved.

### Study design

This is a cross-sectional online survey study in patients who have received PSS/FD care and HCPs who have provided PSS/FD care in Germany, Italy, the Netherlands and Poland. Data collection occurred between April 2023 and May 2024.

### Participants

Patients with any PSS/FD and a wide range of FD diagnoses were included. Eligibility criteria included: (a) scored 10 or higher on the PHQ-15;^19^ (b) sought help for somatic symptoms in the last 12 months; (c) residing and accessing care in Germany, Italy, the Netherlands or Poland; (d) sufficiently proficient in written and oral German, Italian, Dutch or Polish (respectively) and (e) provided informed consent.

Alongside, a range of HCPs were included, including medical specialists, mental health professionals, nurses and other professionals from related fields such as physiotherapy. Eligibility criteria for HCPs were (a) involved in PSS/FD diagnosis/treatment; (b) residing and providing care in Germany, Italy, the Netherlands or Poland; (c) sufficiently proficient in written and oral German, Italian, Dutch or Polish (respectively) and (d) provided informed consent. For the purposes of this study, we focused on those involved in multidisciplinary care.

A convenience sampling approach was used, targeting HCPs caring for patients with current or previous PSS/FD, as well as patient groups. A range of organisations were contacted to share the questionnaire, both for professionals as well as patients. Social media platforms and clinical settings were also targeted, as well as making use of formal and informal contacts through the ETUDE network.

### Questionnaire development and administration

The ARISE online surveys were developed in collaboration with USUMA, specialising in survey design, methodology and analysis. One survey targeting HCPs and one targeting patients with PSS/FD. The surveys were initially drafted in English, with rounds of feedback from USUMA experts to balance validity, completion time and participant experience. To minimise non-response bias and to facilitate participation among individuals suffering from physical symptoms or energy problems, surveys were designed to take no more than 15 min to complete and could be paused and continued at a later time. The surveys were professionally translated into all relevant languages with active verification by team members, patients and HCPs, native to each language to ensure linguistic accuracy and cultural relevance. It was not possible to stop preventing multiple participation, partly as it was important to keep the threshold for participation low and measures to limit multiple participation would have created a barrier to participation, especially for potential participants who may have limited computer literacy. Similarly, it is not possible to report on unique visitor numbers due to the possibility of the same visitor accessing the survey from multiple devices.

### Outcome measures

First, patient participants were asked for demographic information, including age, gender and education level. Education levels were asked based on the International Standard Classification of Education (ISCED) criteria. For clarity, here they are presented in three collapsed categories of low (ISCED 1–2), medium (ISCED 3–4) and high (ISCED 5–8).^20^ Patient participants were also asked whether and what PSS/FD diagnosis they had received and to complete the Patient Health Questionnaire-15 (PHQ-15) as an assessment of their symptom severity.^19^ HCPs were asked about their practice—the number of patients with PSS/FD seen in a month, questions about their profession, healthcare setting, whether they worked in teams and who made up those teams.

The specific measures included in the current study related to experiences of care, primarily multidisciplinary care. The measures included previously existent questionnaires, as well as questions developed for this study. If possible, questions were matched and asked to both patients and HCPs. This was specifically the case for questions on whether participants thought there was sufficient communication between the treating HCPs, and whether the same words and explanations were used by HCPs. Also, a subset of questions on HCP–patient communication from the European Patient Forum 2016 Access Survey^21^ was asked in both groups. Both groups were also asked for the actual and preferred main point of contact (MPC) for patient care. Patients were asked whether the HCP they had most contact with considered issues important to them, and whether they trusted their HCP. HCPs were asked whether all information was accessible by the team and whether there was a clear coordinator of care for each patient. See online supplemental material for the full list of questions (‘Questions’ table).

### Data analysis

No data were missing due to the forced-choice nature of the questions. Sociodemographic differences were explored using χ^2^ or Fisher’s exact test for nominal variables (eg, gender, education level and healthcare setting) and analysis of variance for continuous variables (eg, age, PHQ-15 score and average number of patients with PSS/FD seen per month). Likert scale items were skewed with unequal sample sizes; therefore, they were analysed using the Kruskal-Wallis H test when the four countries were compared. The Mann-Whitney U test was used in the case of comparing Dutch patients with HCPs, as well as identifying differences between pairs of countries when comparing views of patients. Mean ranks and p values are presented for all tests where relevant. A p<0.05 was considered statistically significant. Corrections for multiple comparisons were not done so as not to obscure possible associations, though this does limit the conclusions that can be drawn from the results. Statistical analysis was undertaken using IBM SPSS V.28.

## Results

### Sample characteristics

Overall, 600 patients with PSS/FD were included: 198 from Germany, 174 from Italy, 137 from the Netherlands and 91 from Poland. With regard to HCPs, a total of 152 HCPs from the Netherlands were included, 99 of whom were involved in multidisciplinary care. Exploratory results including HCPs from all four countries can be found in the appendices.

With regard to the patients, 87% were female, with a mean age of 48.4 years (SD=13.3), and a mean PHQ-15 score of 16.0, reflecting a high symptom burden, with higher mean scores in Italy (16.9) and lower scores in Germany (15.2). Polish (63%) and Dutch (52%) patients were most likely educated to a high level, with Italian patients most likely educated to a medium level (40%). A wide range of diagnoses were seen; it is worth noting the high rates of fibromyalgia in Italy (80%) and Poland (52%), and the high rates of irritable bowel syndrome (IBS) in Germany (31%) and the Netherlands (42%). See table 1 for demographic data on patients with PSS/FD.

**Table 1:** Demographics of patients responding to ARISE survey

		DE (n=198)	IT (n=174)	NL (n=137)	PL (n=91)	Total (n=600)	χ^2^/F	P value
Gender—n (%)	Female	172 (87)	156 (90)	112 (82)	80 (88)	520 (87)		0.416
	Male	24 (12)	16 (9)	24 (18)	10 (11)	74 (12)		
	Non-binary	2 (0)	1 (0)	1 (1)	1 (1)	5 (1)		
	Prefer not to answer	0	1 (0)	0	0	1 (0)		
Age	Mean (SD)	47.9 (13.9)	53.5 (11.4)	46.8 (14.1)	42.2 (10.7)	48.4 (13.3)	**F (3)=16.955**	**<0.001**
Education level—n (%)	Low (ISCED 1–2)	59 (30)	50 (29)	23 (17)	6 (7)	138 (23)	**χ2 (6)=38.464**	**<0.001**
	Medium (ISCED 3–4)	61 (31)	69 (40)	43 (31)	28 (31)	201 (34)		
	High (ISCED 5–8)	78 (39)	55 (32)	71 (52)	57 (63)	261 (44)		
PHQ-15	Mean (SD)	15.2 (3.8)	16.9 (4.1)	15.7 (3.5)	16.7 (4.4)	16.0 (4.0)	**F (3)=7.330**	**<0.001**
Received a diagnosis?	N (%)	156 (79)	166 (95)	105 (77)	69 (76)	496 (83)	**χ2 (3)=28.218**	**<0.001**
Diagnosis—N (%)	Chronic pain disorder	53 (27)	14 (8)	8 (6)	11 (12)	86 (14)		**<0.001**
	Fibromyalgia	28 (14)	139 (80)	27 (20)	47 (52)	241 (40)		**<0.001**
	Chronic whiplash syndrome	2 (1)	2 (1)	3 (2)	2 (2)	9 (2)		0.714
	ME/CFS	9 (5)	4 (2)	21 (15)	11 (12)	45 (8)		**<0.001**
	IBS	62 (31)	20 (12)	57 (42)	21 (23)	160 (27)		**<0.001**
	FND	24 (12)	4 (2)	5 (4)	1 (1)	34 (6)		**<0.001**
	Other FD	5 (3)	9 (5)	6 (4)	0	20 (3)		0.091
	Illness anxiety disorder	13 (7)	8 (5)	5 (4)	3 (3)	29 (5)		0.592
	Hypochondriasis	2 (1)	0	0	0	2 (0)		0.525
	BDS	1 (1)	2 (1)	0	3 (3)	6 (1)		0.098
	SSD	10 (5)	1 (1)	8 (6)	0	19 (3)		**0.003**
	Somatisation disorder	8 (4)	0	1 (1)	4 (4)	13 (2)		**0.005**
	Undifferentiated somatoform disorder	2 (1)	0	0	4 (4)	6 (1)		**0.004**
	Somatoform autonomic dysfunction	4 (2)	0	0	2 (2)	6 (1)		0.056
	Single persistent symptom	23 (12)	22 (13)	5 (4)	15 (17)	65 (11)		**0.005**
	Other	61 (31)	34 (20)	47 (34)	19 (21)	161 (27)		**0.008**
	Don't know	5 (3)	1 (1)	0	1 (1)	7 (1)		0.182

Where statistics are not reported, Fisher’s exact was employed. Items in bold are significant.

BDSbodily distress syndromeDEGermanyFDfunctional disorderFNDfunctional neurological disorderIBSirritable bowel syndromeISCEDInternational Standard Classification of EducationITItalyME/CFSmyalgic encephalomyelitis/chronic fatigue syndromeNLThe NetherlandsPLPolandSSDsomatic symptom disorder

Of all the participating HCPs from the Netherlands, 74% were female and had a mean age of 47.4 years (SD=10.5). Of these, HCPs saw on average 14.7 patients with PSS/FD per month (SD=13.9). 56% had more than 10 years’ experience, and 53% worked in outpatient settings. A mixture of professions were included, though mental HCPs represented the most (37% of the total). See table 2 for demographic data on HCPs in the Netherlands (table 5 in supplementary material 1 also provides demographic data on HCPs in all four countries).

**Table 2:** Demographics of Dutch HCPs responding to ARISE survey

		Part of multidisciplinary team (n=99)	Not part of multidisciplinary team (n=53)	Total (n=152)
Gender—n (%)	Female	76 (77)	36 (68)	112 (74)
	Male	22 (22)	17 (32)	38 (26)
	Non-binary	1 (1)	0	1 (1)
Age	Mean (SD)	46.9 (10.6)	48.4 (10.4)	47.4 (10.5)
Years experience	<1 year—total (%)	3 (3)	3 (6)	6 (4)
	1–2 years—total (%)	8 (8)	4 (8)	12 (8)
	3–5 years—total (%)	11 (11)	4 (8)	15 (10)
	5–10 years—total (%)	21 (21)	13 (25)	34 (22)
	>10 years—total (%)	56 (57)	29 (55)	85 (56)
No. of pts seen with PSS/FD in average months	Mean (SD)	15.2 (15.0)	13.8 (11.8)	14.7 (13.9)
Healthcare setting	Inpatient	4 (4)	4 (8)	8 (5)
	Outpatient	53 (54)	28 (53)	81 (53)
	Other	15 (15)	12 (23)	27 (18)
	Mixed	27 (27)	9 (18)	36 (24)
Profession n (%)	GP	12 (12)	7 (13)	19 (13)
	Mental healthcare	35 (35)	21 (40)	56 (37)
	Medical specialist	22 (22)	14 (26)	36 (24)
	Others	28 (28)	8 (15)	36 (24)
	Medical and mental healthcare	0	3 (6)	3 (2)
	Mixed combinations	2 (2)	0	2 (1)

Note on professional categories: The ‘others’ profession represents HCPs with a range of expertise outside of the primary medical specialties. This includes nurses, physiotherapists, speech therapists, dietitians/nutrition counsellors, occupational therapists and social workers. ‘Mixed combination’ refers to participants who reported their professional roles as spanning more than one of the following categories: GP, mental healthcare, medical specialists and other. This excludes those who work exclusively within the combination of ‘medical and mental healthcare,’ which is already a distinct category. Examples of ‘mixed combinations’ include individuals who combine roles such as GP with elements of mental healthcare, or medical specialists who also engage in roles typical of the ‘other’ category.

GPgeneral practitionerHCPhealthcare professional

### Patient experiences of multidisciplinary care

Patients from the Netherlands received less multidisciplinary care than patients from all other countries (NL: 87% vs DE: 92%; IT: 92%; PL: 99%; p=0.010). They also had contact with fewer different disciplines (mean 4.9) than patients from Germany and Poland (6.8 and 7.6 respectively; p≤0.001). With regard to experiences of communication and relationship with their HCPs, patients from Italy stated a better perception of their HCPs, reporting better communication with and between their HCPs, including information about care provided, trust in their HCPs and care adaptations to their needs. The exception to this is that patients in Italy felt less involved in care decisions than patients in the other countries. In contrast, while patients from Poland were less likely than patients from Germany and Italy to have care from multiple disciplines, when multiple disciplines were involved, on average a much higher number of HCPs were involved. They also had a generally poorer experience of the communication between and with professionals than patients in the other countries. See table 3 for the full results of patient experiences of multidisciplinary care.

**Table 3:** Comparison of patients receiving multidisciplinary care with regard to experience of care

		DE	IT	NL	PL	Total	χ^2^/F/H	P value
Had care from multiple disciplines?	Yes–N (%)	190 (92)	163 (92)	123 (87)	91 (99)	567 (92)	**χ2 (3)=11.432**	**0.01**
Number of disciplines involved	Mean (SD)	6.8 (4.2)	5.9 (3.7)	4.9 (3.2)	7.6 (3.7)	6.1 (3.9)	**F (3)=11.125**	**<0.001**
Considered issues important to you?	Mean (SD)	2.1 (0.8)	1.8 (0.9)	2.2 (0.8)	2.0 (0.9)	2.0 (0.8)	**H (3)=21.840**	**<0.001**
Trusted your HCP?	Mean (SD)	1.8 (0.7)	1.6 (0.7)	2.0 (0.8)	2.0 (0.9)	1.8 (0.8)	**H (3)=33.441**	**<0.001**
Sufficient communication?	Mean (SD)	3.0 (0.7)	2.6 (1.0)	2.8 (0.9)	3.1 (0.9)	2.8 (0.9)	**H (3)=29.728**	**<0.001**
Same words/explanations?	Mean (SD)	2.4 (0.8)	2.1 (0.9)	2.6 (0.8)	2.9 (0.8)	2.5 (0.9)	**H (3)=59.018**	**<0.001**
Informed about treatment options	Mean (SD)	3.0 (1.1)	2.9 (1.4)	3.0 (1.1)	3.1 (1.1)	3.0 (1.2)	H (3)=0.998	0.802
Involved in care decisions	Mean (SD)	2.4 (1.1)	2.9 (1.5)	2.5 (1.1)	2.8 (1.4)	2.6 (1.3)	**H (3)=12.725**	**0.005**
Informed about treatment safety	Mean (SD)	2.8 (1.1)	2.0 (1.1)	2.8 (1.1)	2.9 (1.3)	2.6 (1.2)	**H (3)=58.193**	**<0.001**
Care adapted according to needs	Mean (SD)	2.9 (1.2)	2.1 (1.2)	3.0 (1.2)	2.7 (1.3)	2.7 (1.3)	**H (3)=52.429**	**<0.001**
Patient feedback gathered	Mean (SD)	3.5 (1.4)	3.2 (1.3)	3.9 (1.1)	4.1 (1.3)	3.6 (1.3)	**H (3)=37.503**	**<0.001**

For Likert scales, these run from 1 (all the time) to 4 (not at all). Bold values are significant.

DEGermanyHCPhealthcare professionalITItalyNLThe NetherlandsPLPoland

**Figure 1:**
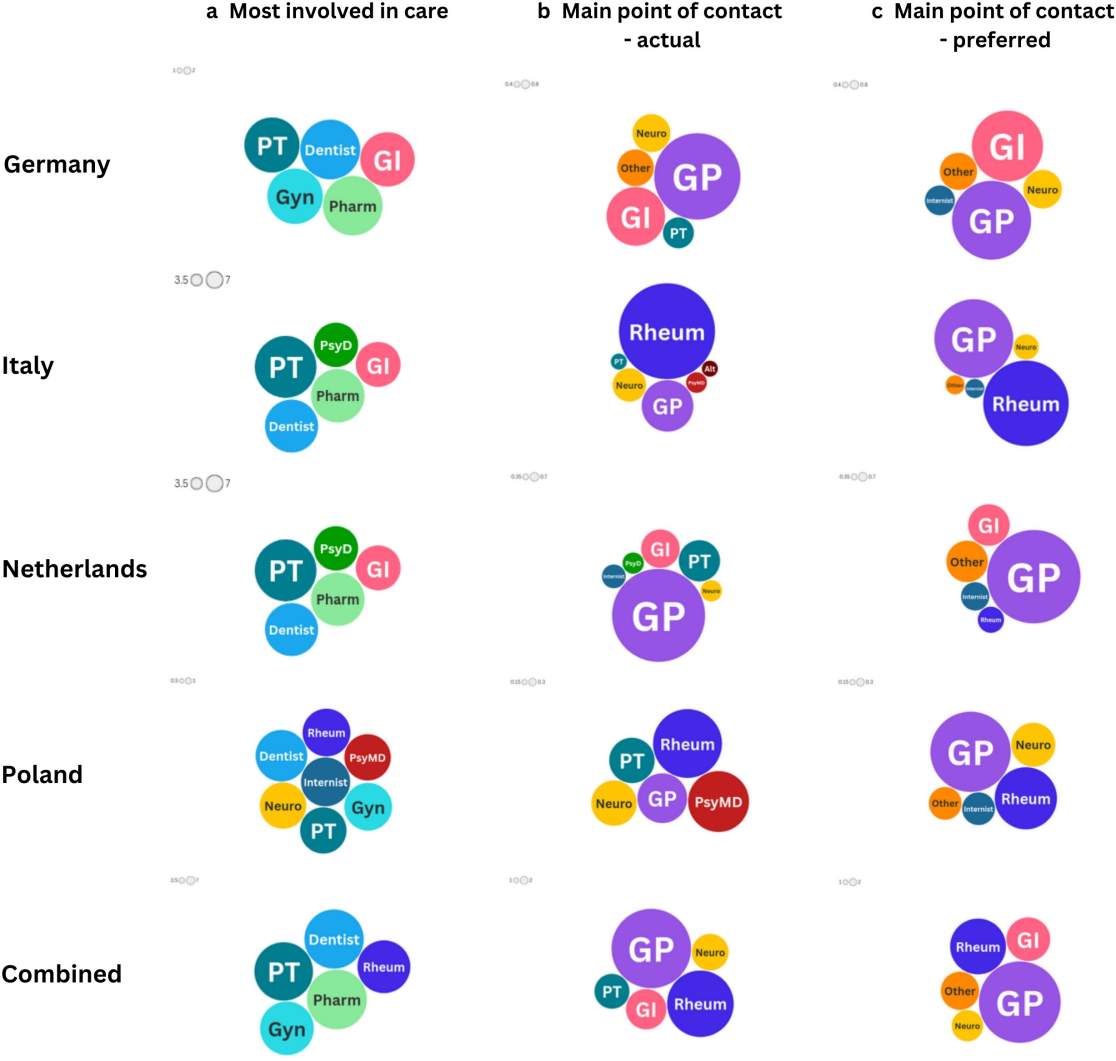
HCPs in patient care—most involved, actual and preferred main point of contact. Comparing patients across different countries. Size of circles denotes number of respondents selecting the specific disciplines relative to the the specific group (eg, main point of contact—actual, for Poland). Alt, alternative/complementary therapist; GI, gastroenterologist; GP, general practitioner; Gyn, gynaecologist; HCPs, healthcare professionals; Neuro, neurologist; Pharm, pharmacist; PsyD, psychologist/psychotherapist; PT, physiotherapist; PsyMD, Psychiatrist; Rheum, rheumatologist.

When asking about HCPs involved in care, GPs were visited by 96% of German, 94% of Dutch and 92% of Italian patients, but only 77% of Polish patients. With regard to the other HCPs involved in patient care, overall, patients reported that pharmacists, dentists and physiotherapists were most commonly involved (8.8%, 8.6% and 8.4% respectively), followed by rheumatologists (7.0%) and gynaecologists (6.8%). Dentists and physiotherapists were among the top five most involved professionals in all four countries, while pharmacists were among the top five in Germany, Italy and the Netherlands. Gynaecologists were among the top five in Germany, Italy and Poland, with rheumatologists being the most involved in Italy, as well as being among the top five in Poland. See figure 1 for the top five disciplines involved in each country alongside the actual and preferred MPC.

With regard to patients’ actual MPC, overall, this has been either GPs or rheumatologists (48.5% and 34.0% overall, respectively), followed by gastroenterologists (12.6%), neurologists (10.3%) and physiotherapists (9.4%). However, there was a lot of variation across the four countries. While GPs, physiotherapists and neurologists were among the top five in all four countries, rheumatologists were only in the top five in Italy (by far the most common MPC) and Poland. Similarly, gastroenterologists were only in the top five in Germany and the Netherlands. Psychiatrists were also commonly the MPC in Poland and Italy, with psychologists commonly the MPC in the Netherlands.

In terms of what patients would prefer, overall, GPs were the most likely to be preferred as the MPC (65.3%), followed by rheumatologists (30.6%), gastroenterologists (18.7%), neurologists (9.6%) and ‘other’ (14.3%). GPs were the most preferred in Germany, the Netherlands and Poland, and the second most preferred in Italy (where rheumatologists were the most preferred). The overall picture of patient preference for the MPC comes down to a preference for GPs or medical specialists, with no country having mental health professionals preferred. This is further supported by the ‘other’ category, which was also in the top five for all countries. The responses in this category referred mostly to specialists in the specific conditions the patient suffered from. Comparing actual and preferred MPC, the main difference lies in the fact that more respondents preferred GPs to be their MPC than was their experience.

### Comparison of patients and HCPs in the Netherlands

**Figure 2:**
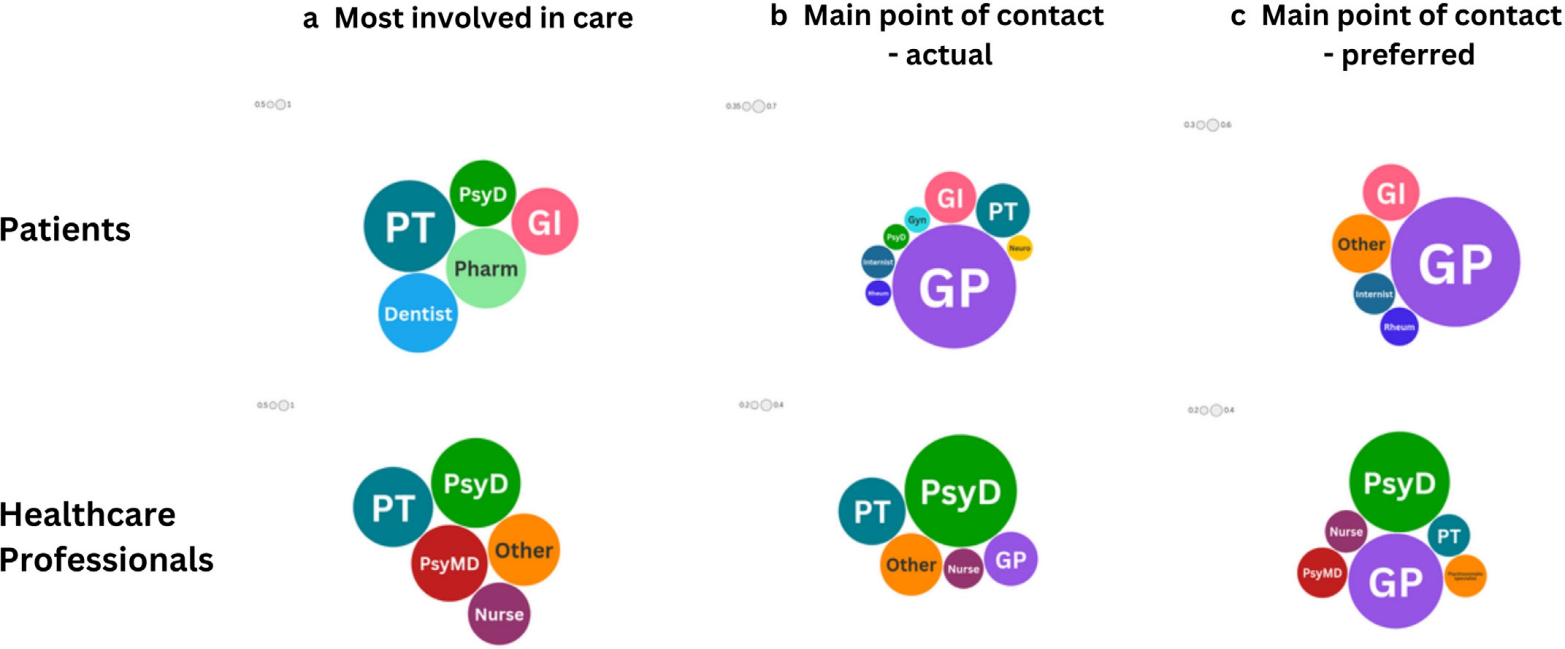
HCPs in patient care—most involved, actual and preferred main point of contact. Comparing patients and HCPS in the Netherlands. Size of circles denotes the number of respondents selecting the specific disciplines relative to the specific group (eg, main point of contact—actual, for patients). Alt, alternative/complementary therapist; GI, gastroenterologist; GP, general practitioner; Gyn, gynaecologist; HCPs, healthcare professionals; Neuro, neurologist; Pharm, pharmacist; PsyD, psychologist/psychotherapist; PT, physiotherapist; PsyMD, Psychiatrist; Rheum, rheumatologist.

Of the patients from the Netherlands, 123 had received care from multiple disciplines, and 99 HCPs stated to have worked in multidisciplinary teams. When comparing the communication experience of patients with HCPs in multidisciplinary care in the Netherlands, we see a significant gap between the two groups. In all areas, HCPs rated their communication better than patients did (see table 4 for details, table 6 in appendix for details on Germany, Italy and Poland). Similarly, a difference is seen in who patients and HCPs thought should be the MPC in patient care (see figure 2). For patients, the preference was clearly for GPs as MPC, selected by 55% of respondents (68/123 respondents). This is followed by ‘other’ (the vast majority of which are disease-specific medical specialists) and gastroenterologists at 14 (11.4%) and 13 (10.6%) respectively, from 123 respondents. While professionals also commonly selected GPs, a much smaller percentage did—26% (25/97 respondents). The HCPs responding were more likely to select psychotherapists/psychologists (29%, 28/97 respondents), which were only selected by three patients (2.5%). Looking at the different professional groups of HCPs, all groups generally selected mental health professionals (in particular psychotherapists/psychologists) and/or GPs, with the exception of GPs who rarely selected themselves (see table 4b in appendix for details).

**Table 4:** Dutch patients who have had care from multiple professionals compared with HCPs who work in multidisciplinary teams

		Patients (n=123)	HCPs (n=99)	U	P value (Z)
Sufficient communication within team?	Mean (SD)	2.8 (0.9)	1.7 (0.7)	**2338.000**	**<0.001 (Z=−8.126**)
Same words/explanations?	Mean (SD)	2.5 (0.8)	1.8 (0.7)	**2936.500**	**<0.001 (Z=−6.570**)
Informed about treatment options	Mean (SD)	2.9 (1.1)	2.1 (0.6)	**3068.000**	**<0.001 (Z=−6.739**)
Involved in care decisions	Mean (SD)	2.4 (1.1)	1.4 (0.5)	**2592.500**	**<0.001 (Z=−7.802**)
Informed about treatment safety	Mean (SD)	2.8 (1.1)	1.9 (0.9)	**3335.500**	**<0.001 (Z=−6.006**)
Care adapted according to needs	Mean (SD)	2.9 (1.1)	1.7 (0.8)	**2432.500**	**<0.001 (Z=−7.968**)
Patient feedback gathered	Mean (SD)	3.9 (1.1)	2.8 (1.3)	**3269.500**	**<0.001 (Z=−6.086**)

For Likert scale, these run from 1 (all the time) to 4 (not at all). Bold values are significant.

HCPhealthcare professional

## Discussion

### Principal findings

A total of 600 patients and 152 HCPs were included in this cross-sectional survey study exploring the experiences of and preferences for multidisciplinary care of patients with PSS/FD. This study compares patients across four European countries with different healthcare systems as well as comparing the experiences and preferences of patients and HCPs from the Netherlands.

Among the differences revealed between patients in the four countries, we found that patients from the Netherlands reported being less likely than all others to have multidisciplinary care, with fewer HCPs involved than patients from Germany and Poland. This may be related to GPs providing a gatekeeping function in the Netherlands. In Poland, on the other hand, fewer patients reported to have received multidisciplinary care than those from Germany and Italy, but when multiple disciplines were involved, more disciplines were involved, possibly related to higher private sector use.^22^ With regard to communication, patients from Italy reported better communication with and between HCPs, including more trust in their HCPs, but felt less involved in decision-making compared with the other countries. Across countries, patients generally reported a preference for GPs, or disease-specific medical specialists, as the MPC.

It was also found that in the Netherlands, patients rated their experience of communication from and between HCPs lower than the HCPs rated the same questions. We do not know if the multidisciplinary care patients reported on was provided within a team-based setting or not, and therefore, whether differences exist in team-based settings. However, these results suggest an overestimation of interdisciplinary communication by HCPs, or an underestimation by patients. With regard to preferences, both patients and HCPs stated a preference for GPs as the MPC. Patients, however, had a much stronger preference for GPs than HCPs, whereas HCPs preferred mental health professionals above GPs. A difference is also seen here between the HCPs involved in care, and their comparative preferences for MPC. Mental HCPs were commonly involved in PSS/FD care, and yet were not among the top-preferred by patients in any of the four countries.

### Strengths and limitations

With regard to strengths, we note first the matched questions allowing for a comparison of patients with PSS/FD across countries as well as being able to compare patients and HCPs. Second, patients with PSS/FD were only involved if they had a certain disease severity, and so we are assessing the experiences and preferences of patients who are more likely to need complex care.

The following limitations are noted in this study. First, HCPs and patients are not matched—we do not assess here the HCPs treating the responding patients. Because of this, we cannot directly compare the views of patients and HCPs in the same service, limiting our ability to draw conclusions on communication differences. Second, while convenience sampling, especially in this case, using easily accessible QR codes and links shared widely online has the advantages of reaching a large number of people, this means that calculating a response rate is not possible. Third, due to this convenience sampling, there is likely a sampling bias as well as differences in recruitment strategies across the four countries, that might explain some of the differences between countries, such as the involvement of mental health professionals in Poland and the Netherlands, versus the involvement of rheumatologists in Italy. This limits the conclusions that can be drawn on the differences. For example, the high rate of fibromyalgia in Italy seems disproportionately high compared with the other countries, which may be a sampling artefact. Fourth, we did not correct for multiple comparisons, and therefore, associations may be overestimated. Finally, we do not know, for the patients’ survey, if the HCPs involved in patient care were specifically involved for the patients’ PSS/FD or for other health needs, and therefore, do not know their background knowledge or affinity to the subject.

### Comparison with the literature

To our knowledge, the ARISE project is the first to look at patients’ and HCPs’ experience of PSS/FD care across different European countries, with no studies in particular comparing the two groups. In other studies looking at general care across Europe, comparisons are generally made with regard to the six cultural dimensions outlined by Hofstede—in particular among these are power distance and individualism.^23^ In certain cases, these dimensions are proposed as the primary causes for differences.^24^ This may explain why there is a significant gap between Italy, and Germany and the Netherlands, with regard to shared decision-making and trust in HCPs in the present study, since Italy has a higher power distance than Germany and the Netherlands.^25^ However, we still see differences in these items comparing Italy to Poland which has an even higher power distance than Italy. Another study^26^ finds that Italians have a more positive attitude towards doctors’ communication than in other countries. Though no hypothesis is proposed to explain this difference, this supports the findings of the present study. The same study does not include any ‘eastern’ European countries (notably Poland), limiting any insights into whether power distance and individualism may be important factors. Studies looking at Polish healthcare do show a lack of trust by patients in their HCPs, but also showing how this may be related to structural challenges in the healthcare system, such as access and resource barriers and long waiting times.^17 27^

### Implications for research and practice

While a gap is noted between the countries with regard to patients’ perception of HCP communication, the reasons for these differences are not entirely clear. A better understanding of the differences can have implications for the organisation of healthcare communication and care coordination. This should specifically include understanding the impact of the different health systems and funding structures on the coordination of care, as well as cultural differences in communication style in the context of multidisciplinary care. Part of this should be to consider the impact multidisciplinary team-based care has on patients with PSS/FD.

In clinical practice, the fact that dentists and pharmacists have so much contact with patients who have PSS/FD seems to be often overlooked. Their involvement in PSS/FD specific care may be limited, but through their contact with patients, they may have an important role to play in communication with patients and HCPs. Improved communication with both of these (and other overlooked) disciplines may help in identifying other areas of care where people suffering from PSS/FD may need additional support.

Another important implication lies in the results of the MPC. The present study and others support GPs and generalists as the MPC (or coordinator of care).^8 12 28 29^ Further studies are needed to understand the reasons behind the selection of GPs by so many, and whether this may relate to their community presence and generalist view. On the other hand, there seems to be a lower selection of GPs as the MPC by GPs themselves. The question of why this is the case, and whether capacity or resource issues play a role should be considered.^29^ It is important to also look at how we can improve coordination of care, for example, by improving the funding and resources available both to primary care and to CCNs in PSS/FD care. Studies could investigate why patients think GPs should be the MPC,^8^ and what it is about GPs and medical specialists that make them preferable to the MPC.

Further research should look into the gap between patients and HCPs in the Netherlands with regard to the experience of communication. Specifically, research should look at patients’ experiences of team-based care, whether this is indeed better than non-team-based care, and whether a gap is still seen in HCPs’ experience of their communication. If a gap is still present, further investigations should look at the factors leading to this.

## Conclusions

This study shows differences in the experiences of communication and multidisciplinary care between patients with PSS/FD across four European countries. Notably, however, it shows similarities in who patients would like to have as their MPC when dealing with their PSS/FD: GPs, or potentially medical specialists. These results are different to what HCPs think should be the MPC, who prefer mental health professionals, followed by GPs. This study also suggests that there may be a gap in the perception of patients and HCPs in the communication from and between HCPs in PSS/FD care. Further research should look at the reasons behind these patients preferences and the differences in experience of communication, towards improving care provided to patients with PSS/FD, both within and across different countries.

## supplementary material

10.1136/bmjopen-2024-097593 online supplemental file 1

10.1136/bmjopen-2024-097593 online supplemental file 2

## Data Availability

Data are available on reasonable request.
