# Supplementary material for: Patient experiences in multidisciplinary care for persistent somatic symptoms across four European countries: a cross-sectional comparison
Source: BMJ Open. 2025 Mar 5;15(3):e097593. doi: 10.1136/bmjopen-2024-097593 (PMC11883617; doi:10.1136/bmjopen-2024-097593)
Supplement: online supplemental file 2 [file bmjopen-15-3-s002.docx]

**Study protocol as available on Open Science Forum - available here:** [**osf.io/rybue**](https://osf.io/rybue)

**Title**

Patient experiences in multidisciplinary care for persistent somatic symptoms across four European countries: a cross-sectional comparison

**Description**

Persistent somatic symptoms (PSS) are conditions characterised by physical symptoms persisting for months, regardless of their underlying cause (Löwe et al., 2022). These conditions affect multiple body systems, and are often influenced by multiple biopsychosocial factors (Löwe et al., 2024). As a result of this, people suffering from PSS require care from multiple professionals and multiple disciplines. In a recent review on functional disorders (FD), services often had seven or eight different disciplines involved in providing care (Mamo et al., 2023). FD are a group of disorders recognised by patterns of PSS. In this review on services for FD, the local context and the conditions cared for likely played a major role in shaping such services.

Unfortunately, the care provided is often fragmented, with patients facing the possibility of long care trajectories, which may at times fail, requiring them to either remain untreated, or start over (Barends et al., 2022, Henningsen et al., 2018). Healthcare professionals from different European countries, which have different healthcare systems, think that better interprofessional collaboration is important for symptom improvement (Kustra-Mulder et al., 2024). This feeds into another study that found a link between the care setting and symptom outcomes. Better outcomes are seen in specialised care settings as opposed to current primary and secondary care (Kustra-Mulder, Löwe & Weigel, 2023). This suggests improving referrals and collaboration between disciplines and between primary, secondary and more specialised care services is important to improve care outcomes.

There are, however, barriers to achieving this which are different in different countries and health systems. From the perspective of healthcare professionals, these include such factors as timely and adequate care barriers, issues with recognition and diagnostic processes, a focus on the biomedical approach, and access and resource challenges (Kustra-Mulder et al., 2024).

However, while we have some understanding of these barriers, and of patients’ broader experiences of accessing PSS care, we do not know much about the experience of patients with regards to multidisciplinary care. Neither do we know much about their preferences when accessing such care, and whether they experienced effective interprofessional collaboration.

Therefore, in this study we aim to explore patients’ experiences of and preferences for multidisciplinary care compare across Germany, Italy, The Netherlands, and Poland. We also aim to compare the experiences of and preferences for multidisciplinary care of patients to those of healthcare professionals. By comparing healthcare across Germany, Italy, the Netherlands and Poland, the results of this study will allow for recommendations to be made for improvements in collaborative care generally and with specific recommendations for these four countries. These recommendations may also form part of future European guidelines on PSS and FD care.

This study uses survey data from the ARISE project ("Health Care Online Survey Europe"). The ARISE project aims to identify aspects of healthcare that may influence the symptom course of individuals with or at risk for persistent somatic symptoms (PSS) and to improve our understanding of healthcare professionals' perspectives on healthcare for individuals with PSS across Europe. We will do this by looking at views on the main point of contact, communication and language use between patients and healthcare professionals within multidisciplinary team, as well as satisfaction of patients with healthcare providers. Further information about the ARISE datasets and studies can be found here: <https://osf.io/rx7t9>, <https://osf.io/3q6hz>.

Research Questions:

1. How do the experiences of and preferences for multidisciplinary care compare between patients across Germany, Italy, The Netherlands, and Poland?
2. How do the experiences of and preferences for multidisciplinary care of patients compare to those of healthcare professionals across Germany, Italy, The Netherlands, and Poland?

**Contributors**

Nick Mamo, Aleksandra Kustra-Mulder, Angelika Weigel, Tim olde Hartman, Lineke Tak, Bernd Löwe, Denise Hanssen, Judith Rosmalen.

**Category**

Project

**Affiliated institutions**

Dimence Alkura, UMCG Department of Psychiatry, UKE Department of Psychosomatic Medicine and Psychotherapy, Radboudumc department of primary care, UMCG Department of Internal Medicine

**License**

CC-By Attribution 4.0 International

**Subjects**

- Health and Medical Administration
- Mental and Social Health
- Medicine and Health Sciences

**Tags**

Multidisciplinary care, PSS, psychosomatic medicine, Europe, patient experience

**Study Information**

Hypotheses

This will primarily be an exploratory study comparing the experiences of and preferences for multidisciplinary care between patients in different countries, and how this compares with healthcare professionals.

1. Experiences of and preferences for multidisciplinary PSS care differ among patients in Germany, Italy, the Netherlands and Poland
2. Experiences of and preferences for multidisciplinary PSS care differ between patients and healthcare professionals

**Design Plan**

Study type

Observational Study

Blinding

- No blinding is involved in this study.

Study design

This will be a cross-sectional survey in patients who have received PSS care and healthcare professionals who have provided PSS care in Germany, Italy, the Netherlands and Poland. We will compare the patients between countries, as well as comparing experiences and preferences of patients with those of healthcare professionals across the four countries.

The data is taken from the ARISE datasets (<https://osf.io/rx7t9>, <https://osf.io/3q6hz>), which are two surveys looking at individuals with PSS and healthcare professionals providing PSS care respectively.

*No files selected*

Randomization

N/A

**Sampling Plan**

Existing Data

Registration prior to accessing the data

Explanation of existing data

The data, whether pre- or post-analysis have not been accessed or viewed by anyone who is defining the research question, or variables under study before registration has been completed.

Data collection procedures

Data collection through convenience sampling was done by targeting relevant groups. In the case of healthcare professionals this was through professional organisations and societies (such as DKPM, EAPM, and WONCA). In the case of patients, this was done through online groups, promotion in clinics and word of mouth. Data was collected through an online survey taking approximately 15 to 20 minutes.

Further details of the data collection procedures for the ARISE datasets are described here: <https://osf.io/rx7t9>, <https://osf.io/3q6hz>.

*No files selected*

Sample size

The sample size for this study is determined by the ARISE datasets. The rationale for these datasets can be accessed here: <https://osf.io/rx7t9>, <https://osf.io/3q6hz>. The data is collected by convenience sampling, aiming for a minimum of 30 participants per country with regards to the healthcare professionals, and 180 participants per country with regards to the individuals with PSS.

Sample size rationale

The sample sizes chosen were to allow for detection of meaningful differences between subgroups. The rationale is described in ARISE dataset registrations here: <https://osf.io/rx7t9>, <https://osf.io/3q6hz>.

Stopping rule

*No response*

**Variables**

Manipulated variables

N/A

*No files selected*

Measured variables

The variables assessed were developed for the surveys. These include the following items:

*Note: Unless specified, the items were assessed using a 4- or 5-point Likert scale.*

For patients:

- Who has been the patient’s main point of contact (multiple choice from a list of possible disciplines)
- Who the patient would prefer to be the main point of contact (multiple choice from a list of possible disciplines)
- Whether the patient thought there was sufficient communication between all professionals involved
- Whether the patient thought all professionals used the same sort of words and explanations
- Whether the healthcare provider considered issues important to the patient
- Whether the patient trusted their healthcare provider they had most contact with
- Whether the treatment received improved symptoms
- How satisfied the patient was with the care of their healthcare practitioner
- A series of five questions were also asked about communication with their healthcare providers. These were adapted from the European Patient Forum (EPF) questionnaire on access to healthcare (European Patients' Forum, 2016).

For healthcare professionals:

- Who has been the main point of contact for patients (multiple choice from a list of possible disciplines)
- Who the professional thinks should be the main point of contact for patients (multiple choice from a list of possible disciplines)
- Whether all relevant information about patients including consultations and results were accessibly by the team involved in the patients’ care
- Whether it is clear who the coordinator of care for individual patients is
- Whether there is sufficient discussion about treatment plans within the team
- Whether all professionals used the same sort of words and explanations throughout the treatment process
- A series of six questions were also asked about communication healthcare providers have with their patients. These were adapted from the EPF questionnaire on access to healthcare (European Patients' Forum, 2016).

We also included demographic questions including age, sex, educational level (in the case of patients) and profession (in the case of healthcare professionals).

*No files selected*

Indices

*No response*

*No files selected*

**Analysis Plan**

Statistical models

We will use a mixture of descriptive statistics, as well as comparison tests – either comparing the four patient groups (across the four countries), or comparing patients with healthcare professionals.

With regards descriptive statistics, we will first describe the populations, in particular with regards to age, sex, education level, and symptoms/illnesses (in the case of patients) and age, sex, and profession (in the case of healthcare professionals). If these analyses reveal important differences between patient populations, we will decide on co-variates and adjustments of the analytical approach described below.

For research question 1, the comparison between the four countries will be made by analysing responses per item. The items on actual and preferred main point of contact will be summarized in a graph and visually analysed; the other items will be analysed using the Kruskal-Wallis H test. For research question 2, the same approach will be used, but then comparing patients and professionals. Based on the descriptive data in the professionals, and the differences between countries in the recruited sample size and professional characteristics, we will decide on which countries provide enough data to analyse the second research question.

*No files selected*

Transformations

*No response*

Inference criteria

*No response*

Data exclusion

*No response*

Missing data

All survey items have so-called forced entry. The forced entry means that if the participants omit or forget to respond to an item, they will get a reminder to fill in the blank spots. They need to fill in the blank spots to move on with the questionnaire. This approach will prevent missing entries in the data. Incomplete surveys will not be included.

Exploratory analysis

*No response*

**Other**

Other

*No response*

***References***

Barends, H., Botman, F., Walstock, E., Dessel, N.C., van der Wouden, J.C., Olde Hartman, T., Dekker, J. & van der Horst, H.E. 2022, "Lost in fragmentation - care coordination when somatic symptoms persist: a qualitative study of patients' experiences", *British journal of general practice,* vol. 72, no. 724, pp. 790–798.

European Patients' Forum 2016, *Access to healthcare EPF's survey -final report*, European Patients' Forum.

Henningsen, P., Zipfel, S., Sattel, H. & Creed, F. 2018, "Management of Functional Somatic Syndromes and Bodily Distress", *Psychotherapy and psychosomatics,* vol. 87, no. 1, pp. 12–31.

Kustra-Mulder, A., Liebau, M., Grewer, G., Rosmalen, J.G.M., Cosci, F., Rymaszewska, J., Löwe, B. & Weigel, A. 2024, "Healthcare Professionals' views on factors influencing persistent somatic symptoms - ARISE-HCP online survey across countries", *Journal of Psychosomatic Research,* , pp. 111695.

Kustra-Mulder, A., Löwe, B. & Weigel, A. 2023, *Healthcare-related factors influencing symptom persistence, deterioration, or improvement in patients with persistent somatic symptoms: A scoping review of European studies*, Elsevier BV.

Löwe, B., Andresen, V., Van Den Bergh, O., Huber, T.B., Von Dem Knesebeck, O., Lohse, A.W., Nestoriuc, Y., Schneider, G., Schneider, S.W., Schramm, C., Ständer, S., Vettorazzi, E., Zapf, A., Shedden-Mora, M. & Toussaint, A. 2022, *Persistent SOMAtic symptoms ACROSS diseases — from risk factors to modification: scientific framework and overarching protocol of the interdisciplinary SOMACROSS research unit (RU 5211)*, BMJ.

Löwe, B., Toussaint, A., Huang, Burton, C., Levenson, J.L., Rosmalen, J.G.M., Huang, W., Weigel, A. & Henningsen, P. 2024, "Persistent physical symptoms: definition, genesis, and management", *The Lancet,* vol. 403, no. 10444, pp. 2649–2662.

Mamo, N., van de Klundert, M., Tak, L., Olde Hartman, T.C., Hanssen, D. & Rosmalen, J. 2023, "Characteristics of collaborative care networks in functional disorders: A systematic review", *Journal of Psychosomatic Research,* vol. 172.
