# Supplementary material for: Patient experiences in multidisciplinary care for persistent somatic symptoms across four European countries: a cross-sectional comparison
Source: BMJ Open. 2025 Mar 5;15(3):e097593. doi: 10.1136/bmjopen-2024-097593 (PMC11883617; doi:10.1136/bmjopen-2024-097593)
Supplement: Supplementary file 3 [file bmjopen-2024-097593.reviewer_comments.pdf]

# PEER REVIEW HISTORY

BMJ Open publishes all reviews undertaken for accepted manuscripts. Reviewers are asked to complete a checklist review form and are provided with free text boxes to elaborate on their assessment. These free text comments are reproduced below.

## ARTICLE DETAILS

**Title (Provisional)**

Patient experiences in multidisciplinary care for persistent somatic symptoms across four European countries: a cross-sectional comparison

**Authors**

Mamo, Nick; Kustra-Mulder, Aleksandra; Hanssen, Denise J.C.; Weigel, Angelika; Tak, Lineke; Olde Hartman, Tim C.; Löwe, Bernd; Rosmalen, Judith GM

---

## VERSION 1 - REVIEW

---

| | |
|---|---|
| **Reviewer** | **1** |
| **Name** | **O'Neal, Mary** |
| **Affiliation** | **Brigham and Women's Hospital, Department of Neurology** |
| **Date** | **01-Jan-2025** |
| **COI** | **None** |

---

Thank you for evaluating this important topic. Your findings are important to consider in how we build multidisciplinary teams for patients with somatization disorders. I think only minor revision is needed.

Please define more clearly:

Somatic symptom disorder, Functional symptoms and FND

---

| | |
|---|---|
| **Reviewer** | **2** |
| **Name** | **Guthrie, Elspeth** |
| **Affiliation** | **University of Leeds Leeds Institute of Health Sciences, Psychological and Social Medicine** |
| **Date** | **06-Jan-2025** |
| **COI** | **None** |

---

This is a very interesting and unusual paper in that it weeks to obtain the perceptions of both patients and HCPs in several different European countries about the management of

functional disorders. The sample of patients (n=600) and HCPs (over 100) is reasonable but obviously not representative of the entire patient population and HCPs in the 4 European Countries. The authors themselves acknowledge this and present a very balanced and measured discussion of the findings. The only point that requires clarification is why the authors chose not to take account of multiple comparisons in their analyses, they used $p < 0.05$ because they said they did not want to 'miss' any potential differences but most of the statistical findings were of the order of $p<0.001$ ?

Apart from this point, this is an interesting and different paper which reflects the differing views across countries and between patients and HCPs of the way functional disorders should be managed.

---

## VERSION 1 - AUTHOR RESPONSE

**Reviewer Reports:**

Reviewer: 1
Dr. Mary  O'Neal, Brigham and Women's Hospital

**Comments to the Author:**

*Thank you for evaluating this important topic. Your findings are important to consider in how we build multidisciplinary teams for patients with somatization disorders. I think only minor revision is needed.*
*Please define more clearly:*
*Somatic symptom disorder, Functional symptoms and FND*

Thank you for your feedback and question. We have provided specific definitions as requested, aside from functional symptoms, which we do not raise as a specific entity within this study. Here is the edited section of the introduction:

“These include conditions such as Fibromyalgia, a disorder of chronic widespread pain [2], Functional Neurological Disorder, a disorder with impairment in voluntary neurological function [3], and somatic symptom disorder, a disorder characterised by persistent somatic complaints alongside disproportionate health-related thoughts, feelings and behaviours in relation to these symptoms [4].”

Reviewer: 2
Dr. Elspeth Guthrie, University of Leeds Leeds Institute of Health Sciences

**Comments to the Author:**

*This is a very interesting and unusual paper in that it weeks to obtain the perceptions of both patients and HCPs in several different European countries about the management of functional disorders. The sample of patients (n=600) and HCPs (over 100) is reasonable but obviously not representative of the entire patient population and HCPs*

*in the 4 European Countries. The authors themselves acknowledge this and present a very balanced and measured discussion of the findings. The only point that requires clarification is why the authors chose not to take account of multiple comparisons in their analyses, they used p< 0.05 because they said they did not want to 'miss' any potential differences but most of the statistical findings were of the order of p<0.001 ? Apart from this point, this is an interesting and different paper which reflects the differing views across countries and between patients and HCPs of the way functional disorders should be managed.*

Thank you for this comment. The choice to use p<0.05 was made before the analyses were performed. The reason was as stated that we did not want to miss any potential differences: We believe that being too strict has the risk to ignore possible associations that could be interesting for future studies. A possible downside of not adjusting for multiple testing, of course, is that maybe we are overestimating the current results or are reporting non-existing associations. That being said, the results showed that most findings were of the order of p<0.001, implying that correcting for multiple test bias would not have altered our conclusions.

---
